# Supplementary material for: Theoretical Study of the Thermal Rate Coefficients of the H3+ + C2H4 Reaction: Dynamics Study on a Full-Dimensional Potential Energy Surface
Source: Molecules. 2024 Jun 12;29(12):2789. doi: 10.3390/molecules29122789 (PMC11206701; doi:10.3390/molecules29122789)
Supplement: Supplementary file 1 [file molecules-29-02789-s001.zip › molecules-3008906-supplementary.pdf]

Theoretical Study of Thermal Rate Coefficients of H_3^+ + C_2H_4 Reaction: Dynamics Study on a Full-Dimensional Potential Energy Surface

Tatsuhiro Murakami [1,2,*], Soma Takahashi [1], Yuya Kikuma [1], and Toshiyuki Takayanagi [1,*]

[1] Department of Chemistry, Saitama University, Shimo-Okubo 255, Sakura-ku, Saitama City, Saitama, 338-8570, Japan
[2] Department of Materials & Life Sciences, Faculty of Science & Technology, Sophia University, 7-1 Kioicho, Chiyoda-ku, Tokyo, 102-8554, Japan
* Correspondence: murakamit@mail.saitama-u.ac.jp; Tel.: +81-48-858-9113 (T.M.); tako@mail.saitama-u.ac.jp; Tel.: +81-48-858-9113 (T.T.)

Supplementary Material

Table. S1. Vibrational frequencies (in cm^{-1}) and zero-point energies (ZPE, in kcal/mol) for the $H_2 \ldots C_2H_5^+$ intermediate complex acquired through machine-learning PES, as well as calculations at the MP2/cc-pVDZ and DF-CCSD(T)-F12a/cc-pVTZ levels.

PES	MP2	DF-CCSD(T)-F12a	
	cc-pVDZ	cc-pVDZ	cc-pVTZ
	$[H_2 \cdots C_2H_5^+]$ (C_{2v})		
147 (A_2)	46 (A_2)	67 (A_2)	67 (A_2)
244 (B_2)	98 (B_2)	98 (B_2)	70 (B_2)
276 (A_1)	136 (B_1)	162 (B_1)	143 (B_1)
317 (B_1)	197 (A_1)	217 (A_1)	228 (A_1)
353 (B_1)	398 (B_1)	420 (B_1)	423 (B_1)
488 (A_2)	763 (B_2)	700 (B_2)	732 (B_2)
789 (B_1)	839 (B_1)	837 (B_1)	839 (B_1)
977 (B_2)	1083 (A_2)	1072 (A_2)	1091 (A_2)
983 (A_2)	1116 (B_1)	1120 (A_1)	1127 (B_1)
1014 (B_2)	1140 (A_1)	1126 (B_1)	1137 (A_1)
1026 (A_1)	1250 (A_2)	1250 (A_2)	1265 (A_2)
1294 (A_1)	1284 (B_2)	1270 (B_2)	1289 (B_2)

1302 (B_1)	1364 (A_1)	1356 (A_1)	1360 (A_1)
1463 (B_2)	1471 (B_2)	1458 (B_2)	1480 (B_2)
1526 (A_1)	1583 (A_1)	1577 (A_1)	1576 (A_1)
2575 (A_1)	2221 (A_1)	2201 (A_1)	2148 (A_1)
3192 (B_2)	3184 (B_2)	3165 (B_2)	3139 (B_2)
3307 (A_1)	3189 (A_1)	3170 (A_1)	3142 (A_1)
3352 (A_2)	3306 (A_2)	3289 (A_2)	3250 (A_2)
3401 (B_1)	3323 (B_1)	3304 (B_1)	3267 (B_1)
4559 (A_1)	4462 (A_1)	4376 (A_1)	4360 (A_1)
ZPE (kcal/mol)			
46.58	46.40	46.08	45.94

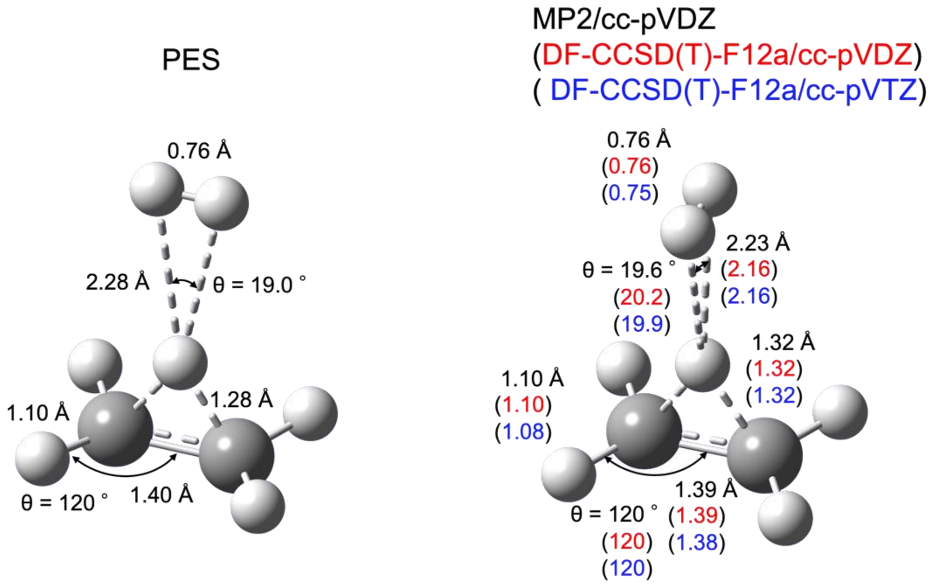

Figure S1. Molecular structure of $H_2 \ldots C_2H_5^+$ intermediate complex with key geometric parameters.

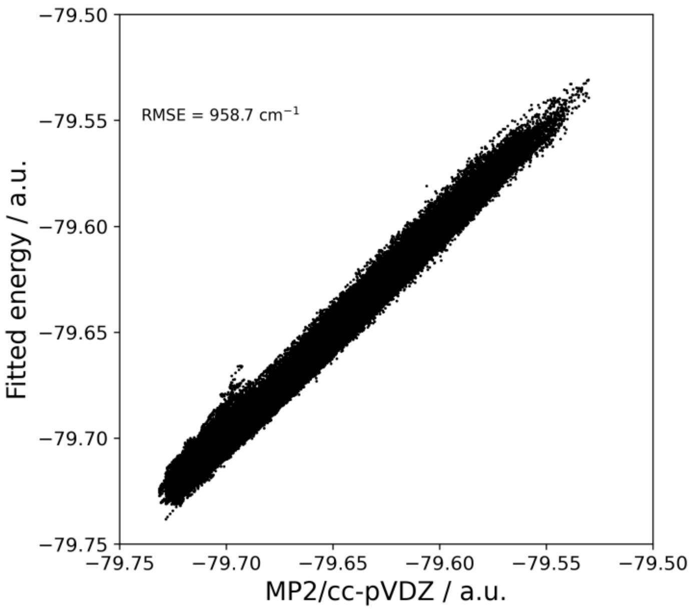

Figure S2. The potential energies fitted based on 330,000 data points computed at the MP2/cc-pVDZ level, yielding a root-mean-square error (RMSE) of 959 cm^{-1}

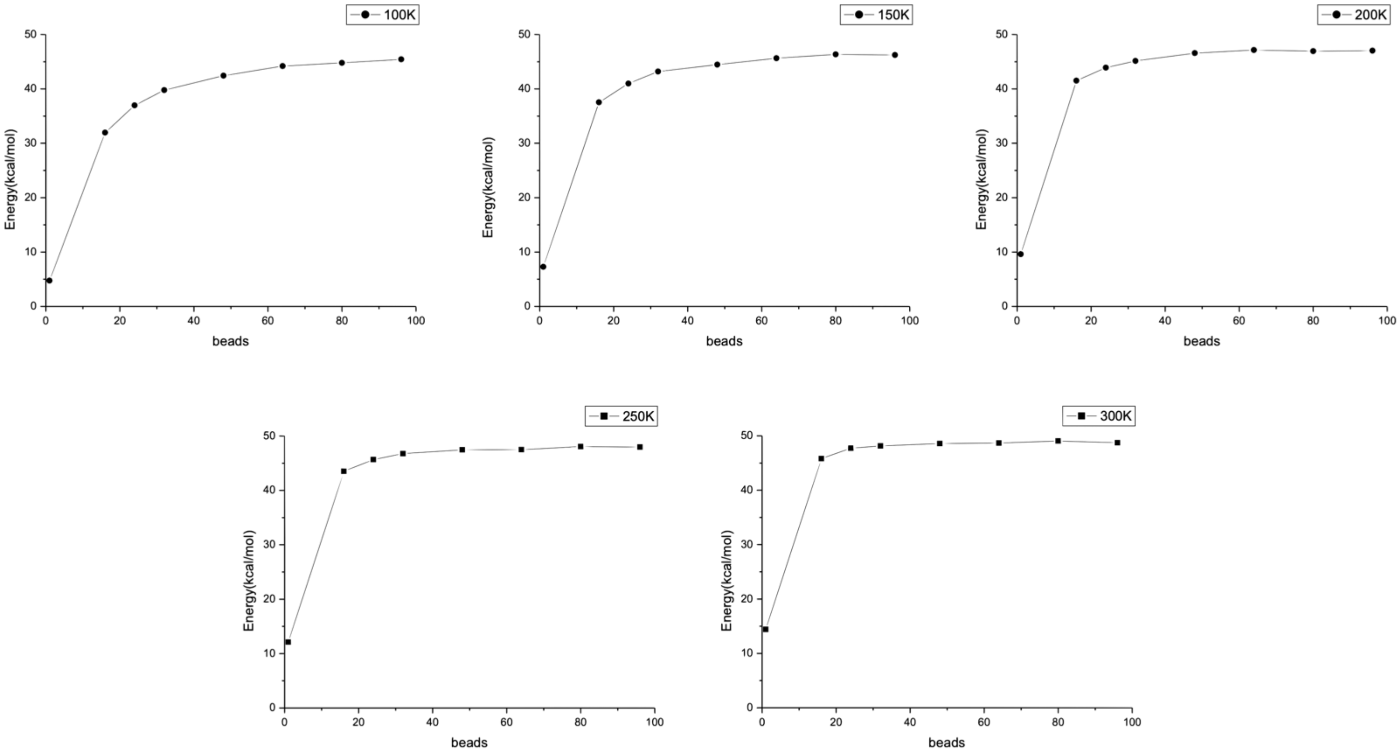

Figure S3. The convergence analysis of bead count concerning internal energies. Note that the zero energy reference corresponds to the potential energy of the $H_2 \ldots C_2H_5^+$ intermediate complex.
